# Fuzzy Decision-Making Fuser (FDMF) for Integrating Human-Machine Autonomous (HMA) Systems with Adaptive Evidence Sources

**DOI:** 10.3389/fnins.2017.00332

**Published:** 2017-06-20

**Authors:** Yu-Ting Liu, Nikhil R. Pal, Amar R. Marathe, Yu-Kai Wang, Chin-Teng Lin

**Affiliations:** ^1^Faculty of Engineering and Information Technology, Center for Artificial Intelligence, University of Technology SydneySydney, NSW, Australia; ^2^Electronics and Communication Sciences Unit, Indian Statistical InstituteCalcutta, India; ^3^United States Army Research Laboratory, Aberdeen Proving GroundAberdeen, MD, United States

**Keywords:** Human-Machine Autonomous (HMA) System, Brain-Computer Interface (BCI), Fuzzy Decision-Making Fuser (FDMF), Dempster-Shafer Theory, Information Fusion

## Abstract

A brain-computer interface (BCI) creates a direct communication pathway between the human brain and an external device or system. In contrast to patient-oriented BCIs, which are intended to restore inoperative or malfunctioning aspects of the nervous system, a growing number of BCI studies focus on designing auxiliary systems that are intended for everyday use. The goal of building these BCIs is to provide capabilities that augment existing intact physical and mental capabilities. However, a key challenge to BCI research is human variability; factors such as fatigue, inattention, and stress vary both across different individuals and for the same individual over time. If these issues are addressed, autonomous systems may provide additional benefits that enhance system performance and prevent problems introduced by individual human variability. This study proposes a human-machine autonomous (HMA) system that simultaneously aggregates human and machine knowledge to recognize targets in a rapid serial visual presentation (RSVP) task. The HMA focuses on integrating an RSVP BCI with computer vision techniques in an image-labeling domain. A fuzzy decision-making fuser (FDMF) is then applied in the HMA system to provide a natural adaptive framework for evidence-based inference by incorporating an integrated summary of the available evidence (i.e., human and machine decisions) and associated uncertainty. Consequently, the HMA system dynamically aggregates decisions involving uncertainties from both human and autonomous agents. The collaborative decisions made by an HMA system can achieve and maintain superior performance more efficiently than either the human or autonomous agents can achieve independently. The experimental results shown in this study suggest that the proposed HMA system with the FDMF can effectively fuse decisions from human brain activities and the computer vision techniques to improve overall performance on the RSVP recognition task. This conclusion demonstrates the potential benefits of integrating autonomous systems with BCI systems.

## Introduction

Human variability (due to fatigue, concentration lapses, or disorientation) and environmental uncertainties (caused by complexity, danger, or unexpected disturbances) pose serious challenges to traditional brain-computer interface (BCI) systems in real-life applications (Nijboer et al., 2010; Guger et al., 2012). Human variability arises because cognitive states of individuals continually change over time due to factors such as fatigue, attention, and stress. Dynamic environments may lead to significant changes in the performance of BCI systems because individuals have different capacities to adapt to environmental changes. One potential avenue for improving BCIs is by integrating BCI technologies with autonomous or intelligent systems to enhance the performances of joint human-autonomy tasks (Kapoor et al., 2008; Pohlmeyer et al., 2011; McMullen et al., 2014). Such enhancement requires automated decision fusion methods that can mitigate the enormous volume of human or machine information that could otherwise overload human analysts.

Decision making is crucial in a number of industrial applications, including control, dynamic system identification, spatiotemporal pattern recognition, forecasting, and bioengineering, that involve many intricate factors (e.g., prior knowledge, data characteristics, and systematic variation). To enhance the utility of each decision, a decision is often made by considering all the alternatives. In many multiple-attribute decision-making (MADM) problems (Hu et al., 2006; Deng and Chan, 2011), decision makers are usually required to employ a set of alternatives or options in which each option may depend on a range of both quantitative and qualitative information. Therefore, an effective framework for integrating such multi-modal information is an important issue. A standard rationale of decision making is to assess the probabilities with which each consequence is reaped from the selection of individual actions—it is an approach that provides a quantifiable evidence to indicate the most desirable action. The selected action (i.e., the final decision), is the one with the highest utility under the given circumstances and the available information. However, since each decision is associated with different levels of uncertainty, it is essential to establish a reliable decision-support system with a flexible framework that can represent both qualitative and quantitative uncertainty for each possible evidence source.

Recently, many attempts have been made to integrate techniques from machine learning and statistical fields to handle uncertain information (Hu et al., 2006; Wu, 2009; Deng and Chan, 2011; Luo and Lai, 2014; Yager and Alajlan, 2014). Because of the flexibility with which uncertain information can be dealt with, it is reasonable to process MADM problems using fuzzy sets theory (Zadeh, 1965) and Dempster-Shafer (D–S) theory (Dempster, 1967; Shafer, 1976). Hu et al. (2006) proposed a general scheme for attribute aggregation in MADM under uncertainty using an evidential reasoning approach based on the D–S theory. In another study (Wu, 2009), Wu utilized gray-related analysis and D–S theory to address fuzzy group decision-making problems for supplier selection. Wu (2009) applied the D–S combination rule to gather individual preferences into a collective preference through group aggregation. A D–S belief structure was developed by Casanovas and Merigó (2012) to solve the decision-making problem when the available information is in the form of fuzzy numbers.

Obviously, most real-world knowledge is fuzzy rather than precise, and often, real-world decision-making problems that can be handled easily by humans are too difficult for machines to handle. The Dempster–Shafer theory (D–S theory) of evidence enables us to integrate heterogeneous information from multiple sources to obtain collaborative inferences for a given problem.

One of the major advantages of D–S theory is that it provides a straightforward, yet useful, way to quantify ignorance (non-specificity) and conflict (randomness); therefore, it is a suitable framework for handling incomplete uncertain information.

This study proposes an HMA system that simultaneously aggregates human and machine knowledge to recognize targets that appear in a rapid serial visual presentation (RSVP) task. RSVP is one kind of oddball paradigm. P300 wave is an event-related potential (ERP) that is generated as a response to the infrequent target stimulus shown in RSVP sequences and majorly observed in the posterior area (Lin et al., 2015). Thus, detection of reaction to a low-probability target in RSVP would trigger P300 wave, which we extracted in this study. The HMA system dynamically aggregates decisions that include uncertainties from both human and autonomous agents. These uncertainties arise because the characteristics of prediction models can severely influence the reliability of different decisions made by both agents. A primary goal of the HMA is to exploit the capabilities of human and autonomous machines to achieve and maintain a better performance more efficiently and robustly than either the human or the autonomous agents can achieve independently.

To accomplish this, a fuzzy decision-making fuser (FDMF) is proposed in this study that provides a natural adaptive framework for evidence-based inference by incorporating an integrated summary of the available evidence and associated uncertainty. The kernel of the FDMF is based on a multi-attribute (i.e., various human and machine information) evaluation framework and Dempster's rule of combination in the D–S evidence theory. The HMA system involves two steps: (1) detecting targets via human and autonomous systems and (2) integrating decisions from different agents using the FDMF for human-autonomy interaction.

## Human-machine autonomous system

**Figure 1:**
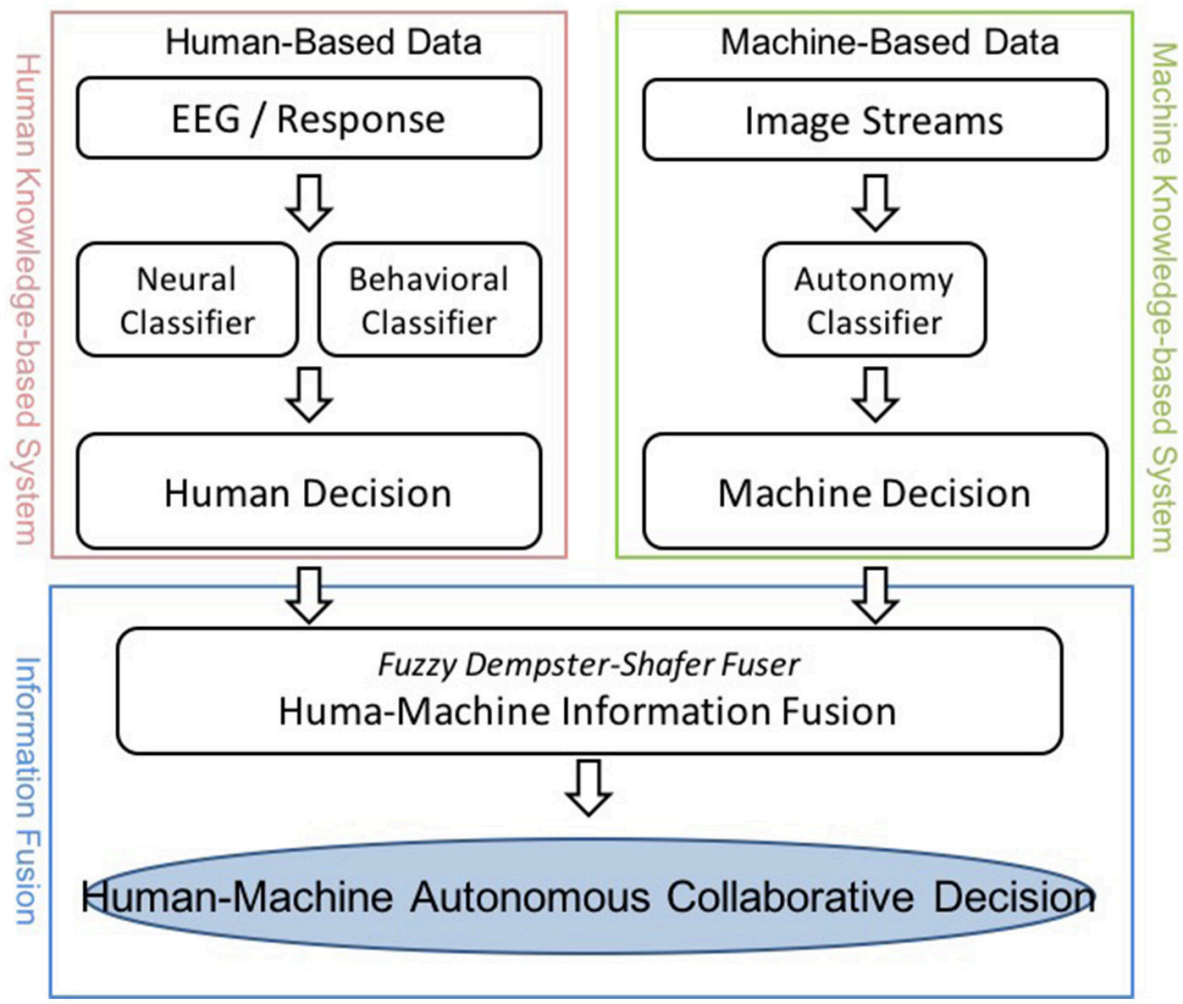
The proposed Human-Machine Autonomous System.

**Figure 2:**
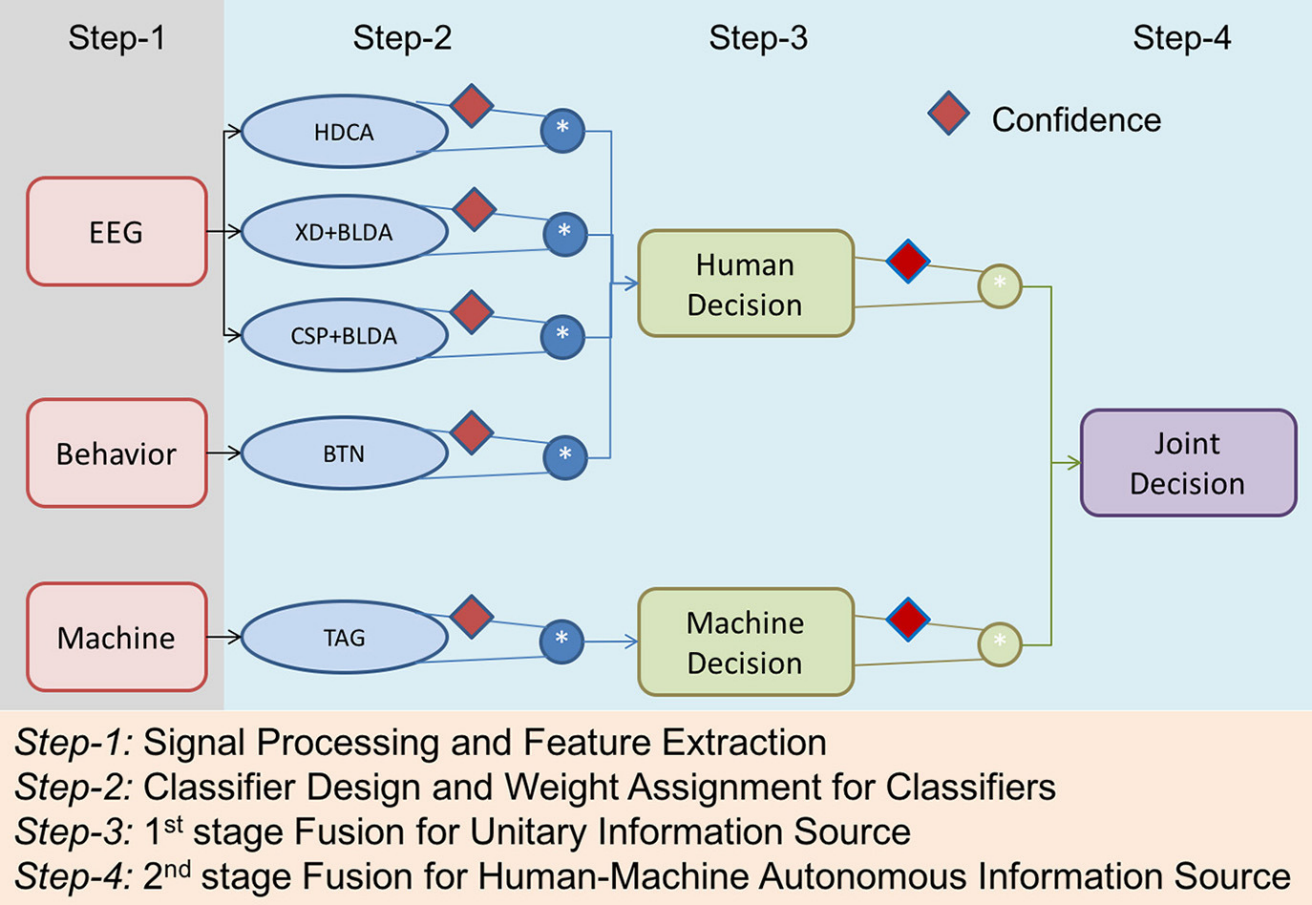
Flowchart of the Human-Machine Autonomous System.

Numerous approaches have been proposed to develop human-machine integration systems (Marathe et al., 2014; Vogel et al., 2015; Wang et al., 2015). Figure 1 depicts the infrastructure of an envisioned HMA system that comprises a human and a machine knowledge-based system. In this study, the specific task exploited the RSVP paradigm. The human knowledge for the experiments was collected via behavioral and physiological observations from a recent RSVP study (Touryan et al., 2014), and the machine knowledge-based system was established using an established computer vision algorithm based on the semi-automated Transductive Annotation by Graph (TAG) system (Wang and Chang, 2008). Figure 2 shows a complete flowchart of the HMA system. We will describe details of each block the following sections.

### Human knowledge-based system

BCIs can determine user intent from different electrophysiological signals (such as EEG). For instance, the user may control brain wave modulation (e.g., mu or beta rhythms, Pfurtscheller et al., 2010; Wu et al., 2016) or the BCI may exploit the natural automatic brain responses to external stimuli (e.g., event-related potentials, Liu et al., 2016). The human knowledge-based system proposed in this study is based on BCI technology.

In this study, EEG recordings of brain activity were utilized to measure each participant's brain dynamics during the RSVP task. The EEG signals were captured from 256 active electrode sites. All the EEG electrodes were placed in accordance with the standard 10–10 system of electrode placement. To reduce noise in the measured EEG signals, the contact impedance between the EEG electrodes and the cortex was calibrated to be <5 kΩ before recording. The EEG data were recorded using a BioSemi ActiveTwo system (Amsterdam, Netherlands). During the recording process, the subjects were instructed to press a button as they discovered a target object in the RSVP stream. The response time (RT) of each subject represented the time interval between the appearance of the object and pressing of the button. The participant's response was calculated and recorded during the experiment. EEG signals were recorded at a sampling rate of 1,024 Hz, in accordance with the hardware specifications. Using the EEGLAB toolbox (Delorme and Makeig, 2004), the EEG data were downsampled to 250 Hz, digitally band-pass filtered between 0.5 and 50 Hz, and down-selected to the 64 channels that most closely matched the standard 10–10 EEG electrode arrangement. These preprocessed data from the 64 channels were then segmented into epochs lasting from 500 ms before to 1,000 ms after the appearance of each image, and the epoched data served as inputs for the neural classification algorithms.

Three existing neural classification algorithms were adopted to recognize target from non-target images based on the neural responses to each image. Note that, here neural classifier does not refer to neural networks, but a classifier that uses neural responses. The three classification algorithms were: (1) hierarchical discriminant component analysis (HDCA; Gerson et al., 2006), which consists of a two-layer ensemble method that uses linear discriminant classifiers to differentiate targets from non-targets based, first, on the spatial and then on the temporal distribution of neural activities; (2) XDAWN, which uses an unsupervised method to estimate spatial filters that enhance the P300 evoked potential by maximizing the signal to signal plus noise ratio (Rivet et al., 2009; Cecotti et al., 2011). It then uses a Bayesian Linear Discriminant Analysis (XD+BLDA) as the classifier; and (3) Common Spatial Patterns (CSP), which is based on a spatial filtering method that identifies the linear combinations of the neural signals which maximize the variance between the target and non-target responses (Ramoser et al., 2000), which were then classified using BLDA. The behavioral classifier (BTN) was constructed based on the RT between the image onset and button press.

### Machine knowledge-based system

The machine knowledge-based system used in this study was based on a semi-automated TAG system (Wang and Chang, 2008), which estimates the posterior probability that a specific image contains a target object based on a limited number of labeled bounding boxes drawn around target and non-target objects. Because each of the five object classes (further details are available in Section Rapid Serial Visual Presentation Paradigm) was designated as the target for at least one block of trials during the RSVP task, one classifier was trained for each image class (object class). The feature vectors consisted of dense scale-invariant feature transform (SIFT) features (Lowe, 1999; Bosch et al., 2006) extracted from each image and then clustered and quantified using a codebook in a “bag-of-words” approach (Csurka et al., 2004). Potential bounding boxes in the unlabeled images were identified using “objectness” detectors, which found 25 boxes in each image with the greatest probability of containing a well-defined object (Alexe et al., 2012), resulting in a target probability for each bounding box in each image. The largest target probability of any bounding box in a given image became the computer vision (CV) score for that image.

Both human and machine knowledge systems yielded a final score between 0 and 1 for each image. A score closer to 1 indicated that the image was likely to contain a target object, while a score closer to 0 indicated a non-target image. Because the score was not a binary value, a threshold was used to divide the continuous values into the two categories. To identify an appropriate threshold for discriminating targets from non-targets, output scores were generated for two independent datasets. The training set was used to find the threshold value that maximized the difference between true positive and false positive rates using the Receiver Operating Characteristic (ROC) curve. Then, the testing set used this threshold to make the final binary decision.

### Fuzzy decision-making fuser

**Figure 3:**
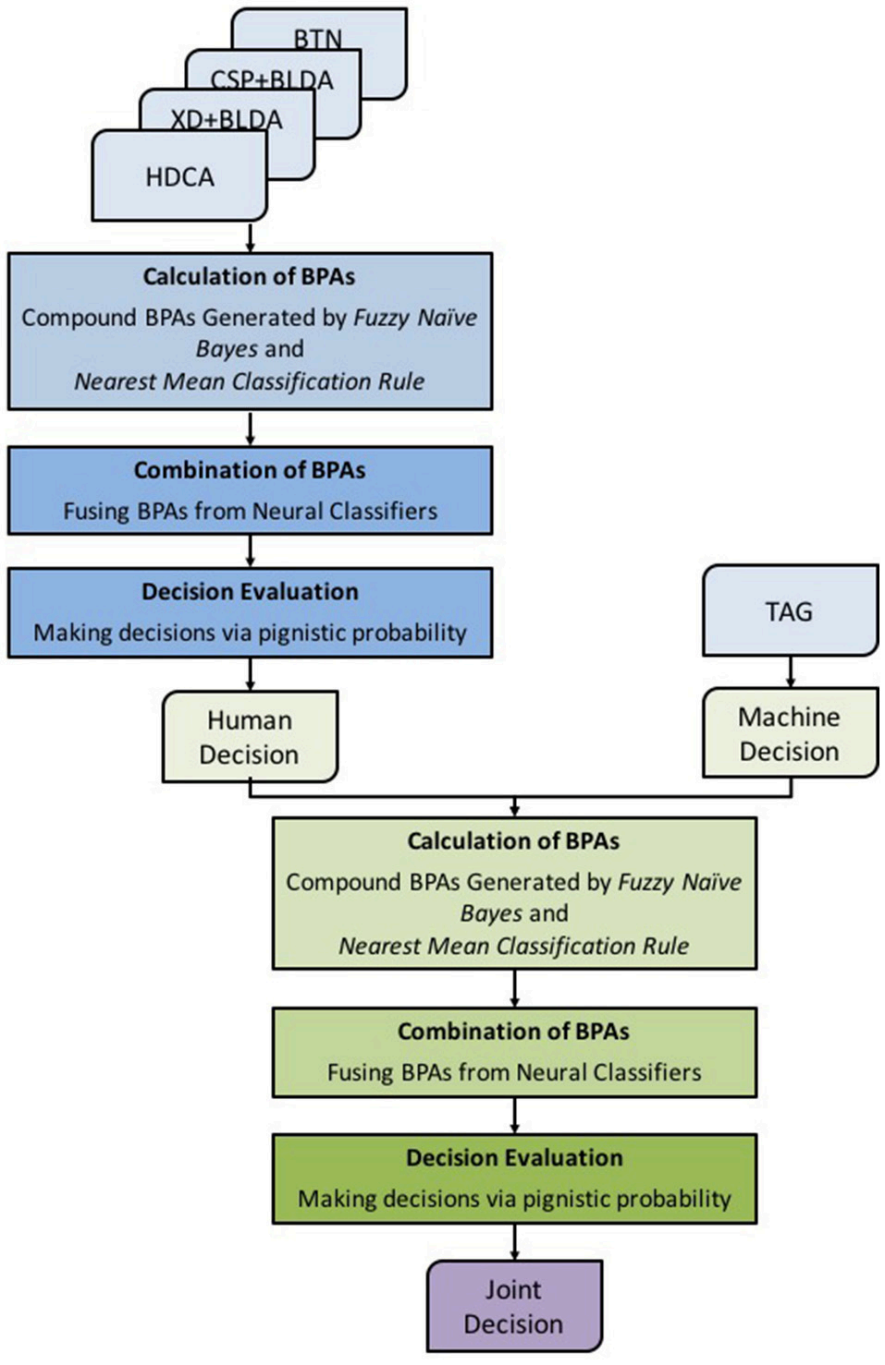
Framework of the Fuzzy Decision-Making Fuser.

To efficiently integrate decisions/classifier outputs from different human and machine agents, we propose to use an FDMF to model and analyze the HMA system with multiple inputs and outputs. The FDMF framework is shown in Figure 3. The FDMF kernel is established based on D–S theory, which provides a mechanism for representing and processing uncertain, imprecise and incomplete information from human, and machine agents (Liu et al., 2017). Furthermore, the final decision is made using Dempster's rule of combination (Dempster, 1967; Shafer, 1976) to integrate the information/evidence from different sources. The FDMF utilizes a novel compound model that combines generative-type and discriminative-type approaches to determine basic probability assignments (BPAs). Unlike most other methods, the proposed method applies a plausible mathematical structure to determine the weights of evidences. In addition, an intuitionistic belief assignment is employed to capture uncertainties between classes, which can directly represent uncertainties and imprecision during the classification process. Generative-type BPAs are often associated with probability distributions, fuzzy membership or possibility functions to address recognition problems. In the proposed method, the generative-type BPA of an incoming datum to each class is assigned based on a fuzzy Naïve Bayes approach (Tang et al., 2002) using the degree of fuzzy membership to each category. In contrast, discriminative-type BPAs are built on the concept of similarities of a training pattern to class-representative vectors or vectors representing class boundaries. These similarities are often referred to as distances, specificity, or consistency. The nearest mean classification (NMC; Veenman and Reinders, 2005) rule is employed to determine discriminative-type BPA in this study. We utilized the distance of a test data point from the nearest class mean to define the discriminative-type BPA.

First, we consider assignment of generative type BPA on singletons. Let *X*_*i*_, *i* = 1…*p* be *p* independent variables (features), and let each object, *X*, be represented by a *p*-dimensional feature vector. Let *Y* be the class label associated with *X*; *Y* ∈ *C* = {*C*_1_, *C*_2_, …, *C*_*N*_}. To determine a generative-type BPA, the fuzzy c-means clustering algorithm is used to find a set of *c* clusters that results in an appropriate fuzzy partition of the domain of each variable. Consequently, the class conditional probability to class *C*_i_ decided by the fuzzy Naïve Bayes approach (Tang et al., 2002) is assigned as the basic probability that is used in the architecture of D–S theory:

(1) $$\begin{array}{r} m\left(C_{i}\right)=\mu_{C_{i}}\left(x\right). \end{array}$$

We emphasize here that, although we are calling the assignment in Equation (1) a BPA, the result may not be a valid BPA because the sum of assignments might not be equal to 1 as demanded by D–S theory. Later, we will use a normalization scheme to define a valid BPA.

To assign the discriminative-type basic probability on singletons, the centroid vectors of all classes can be calculated and denoted by {*v*_1_, *v*_2_, …, *v*_*N*_}. An incoming sample may then be classified according to the minimum Euclidean distance from the class centroids.

The square of the Euclidean distance is computed by

(2) $$\begin{array}{r} d\left(x^{s},V_{i}\right)={\left(x^{s}-V_{i}\right)}^{T}\left(x^{s}-V_{i}\right). \end{array}$$

In the D–S formalism, the mass function *m*(.) can be defined using an exponential function over the Euclidean distance as follows:

(3) $$\begin{array}{r} m\left(C_{i}\right)=e^{-d\left(x^{s},V_{i}\right)}. \end{array}$$

However, in the present investigation, although we use the same principle, we define BPAs using each attribute separately. We do so because we want to exploit class-specific discriminative properties of different attributes, which is lost if we use distance between vectors to define BPAs.

Next, we consider the assignment of mass to compound hypothesis that focuses on sets with cardinality more than one. Let us denote Θ as the frame of discernment (Dempster, 1967; Shafer, 1976):

(4) $$\begin{array}{r} \Theta =\left\{Target,NonTarget\right\}. \end{array}$$

The focal elements of the power set of the frame of discernment, 2^Θ^, are denoted by

(5) Ω={{Target},{NonTarget},{Target,NonTarget},

where the compound element {*Target, NonTarget*} is an uncertain hypothesis in the D–S formalism.

Unlike previous studies (Denoeux, 1995, 2000; Pal and Ghosh, 2001), which used the complementary concept to determine BPAs for compound hypotheses, this study proposes a rational way to define the center of the Region of Uncertainty (ROU) based on the threshold value that maximizes the difference between the true positive and false positive rates in each classifier, and uses that threshold to assign mass to focal sets with cardinality more than one. That is, clearly, the samples that are closer to the center of the ROU will be difficult to discriminate because they possess properties of two different classes (e.g., Target and Non-Target) *simultaneously*. Specifically, samples falling in the ROU are likely to result in classification errors in the recognition task. Thus, it is natural to represent the ROU by the compound hypothesis {*Target, NonTarget*}. The threshold value that maximizes the difference between the true positive and false positive rates in each classifier is exploited as the centroid of the compound hypotheses {*Target, NonTarget*}.

For each decision agent, fuzzy membership functions {μ_{*Target*}_, μ_{*NonTarget*}_} are obtained to represent the degree of membership of each object to different classes when represented by independent individual attributes. Given an incoming sample, for feature *x*, the calculated membership-value using the Fuzzy Naïve Bayes Approach (recalling Equation 1) is

(6) $$\begin{array}{r} m_{x}^{g}\left(\left\{C_{i}\right\}\right)=\mu_{\left\{C_{i}\right\}}\left(x\right), \end{array}$$

where *C*_*i*_ ∈ {*Target, NonTarget*}.

Because with a compound hypothesis, {*Target, NonTarget*}, an object may belong to either class *Target* or class *NonTarget* (but it possesses properties of *both Target* and *Non-Target*), a fuzzy AND operator is used to assign the mass associated with {*Target, NonTarget*}. Thus, the generative basic probabilities of different hypotheses calculated by the fuzzy Naïve Bayes approach are defined as follows:

(7) $$\begin{array}{l} \;m_{x}^{g}\left(\left\{C_{i}\right\}\right)=\mu_{\left\{C_{i}\right\}}\left(x\right)\\ m_{x}^{g}\left(\left\{Target,\;NonTarget\right\}\}\right)=\mu_{\left\{Target,\;NonTarget\right\}}\left(x\right)\\ \;=\mu_{\left\{Target\right\}}\left(x\right)\wedge \mu_{\left\{NonTarget\right\}}\left(x\right). \end{array}$$

Again, we note that $m_{x}^{g}$ as calculated by Equation (7) may not lead to a valid BPA without proper normalization. In Equation (7), one can use any t-norm for ∧ (Dubois and Prade, 1985); however, in this study, we use minimum as the t-norm.

Next, the component wise Euclidean distance between the incoming sample and the centroid vector {*v*_{*Target*}_, *v*_{*NonTarget*}_} of each class is utilized to determine the discriminative basic probability according to the NMC rule. Then, we use the ROU as the support for the compound hypothesis, and define the crossover point *v*_{*Target, NonTarget*}_ for each attribute as the centroid to compute the mass for the compound hypothesis, where *v*_{*Target, NonTarget*}_ lies in the interval [*v*_{*Target*}_, *v*_{*NonTarget*}_] —this crossover point has the maximum uncertainty.

One possible way to define the discriminative BPAs is to use an exponential function of distances from the NMC as follows:

$$\begin{array}{l} m_{x}^{d}\left(\left\{C_{i}\right\}\right)={k_{1}e}^{-k_{2}d\left(x,V_{i}\right)};k_{1},\;k_{2}>0 \end{array}$$

(8) $$\begin{array}{r} m_{x}^{d}\left(\left\{Target,\;NonTarget\right\}\right)=k_{1}e^{-k_{2}d\left(x,v_{\left\{Target,\;NonTarget\right\}}\right)}. \end{array}$$

Note that, Equation (8) defines the discriminative type BPA for an attribute, and hence *x* is the value of an attribute (say the k^th^ attribute) and *V*_i_ is the k^th^ component of the *i*th centroid representing the i^th^ class. For simplicity, in this study we have used *k*_1_ = *k*_2_ = 1. Furthermore, we use a weighted regulatory architecture to integrate discriminative and generative types of evidence (Liu et al., 2016). The generative-type BPAs, $m_{x}^{g}\left(\left\{\cdot \right\}\right)$, generated by the fuzzy Naïve Bayes approach and the discriminative-type BPAs, $m_{x}^{d}\left(\left\{\cdot \right\}\right)$, calculated by the NMC rule are then integrated as follows:

(9) $$\begin{array}{r} \phi_{x}\left(\left\{\cdot \right\}\right)={m_{x}^{g}\left(\left\{\cdot \right\}\right)}^{\alpha }{m_{x}^{d}\left(\left\{\cdot \right\}\right)}^{\beta }, \end{array}$$

where 0 ≤ α, β ≤ 1 are regulatory parameters that adaptively determine the importance of the two types of evidence. The weighted regulatory mechanism enables us to use the training data to search for the appropriate weights for different sources of evidence. The optimal values of the regulatory parameters are found using grid search by minimizing the training error. Note that $m_{x}^{g}$ and $m_{x}^{d}$ are not BPAs *per se* because the sum of the basic assignments may not be equal to one. However, in the combined BPA mentioned below we enforce this condition.

The compound BPA for feature *x*, *m*_*x*_({·}) is defined as follows:

$$\begin{array}{l} m_{x}\left(\left\{C_{i}\right\}\right)=\frac{\phi_{x}\left(\left\{C_{i}\right\}\right)}{L}=\frac{{\left(\mu_{\left\{C_{i}\right\}}\left(x\right)\right)}^{\alpha }\cdot {\left(e^{-d\left(x,v_{\left\{C_{i}\right\}}\right)}\right)}^{\beta }}{L} \end{array}$$

(10) $$\begin{array}{l} m_{x}\left(\left\{Target,\;NonTarget\right\}\right)=\frac{\phi_{x}\left(\left\{Target,\;NonTarget\right\}\right)}{L}\\ =\frac{\begin{array}{c} {\left(\mu_{\left\{Target,\;NonTarget\right\}}\left(x\right)\right)}^{\alpha }\cdot \\ \;{\left(e^{-d\left(x,v_{\left\{Target,\;NonTarget\right\}}\right)}\right)}^{\beta } \end{array}}{L} \end{array}$$

where *L* is a normalizing factor to ensure that Equation (10) is a valid BPA.

(11) $$\begin{array}{r} L=\sum_{i=1}^{N}\phi_{x}\left(\left\{C_{i}\right\}\right)+\sum_{i=1}^{N}\sum_{j>i}^{N}\phi_{x}\left(\left\{C_{i},C_{j}\right\}\right) \end{array}$$

In the present investigation, *N* = *2*, and *C*_1_ = Target and *C*_2_ = Non-Target. For each attribute (here each classifier/agent), using Equations (9)–(11), we get one compound BPA. These compound BPAs generated from the different decision agents/classifiers (attributes) are then combined to get an overall BPA using Dempster's rule of combination (Shafer, 1976). Let *m*_1_ and *m*_2_ be two items of evidence introduced by two independent sources. Dempster's rule of combination (Dempster, 1967; Shafer, 1976), which is denoted as *m* = *m*_1_ ⊕ *m*_2_ within the framework of evidence theory, integrates the two BPAs, *m*_1_ and *m*_2_, to yield a combined BPA as follows:

(12) $$\begin{array}{r} m_{1}⊕m_{2}\left(A\right)=\frac{1}{1-\kappa }\sum_{B\cap C=A}m_{1}\left(B\right)m_{2}\left(C\right) \end{array}$$

(13) $$\begin{array}{r} \kappa =\sum_{B\cap C=\phi }m_{1}\left(B\right)m_{2}\left(C\right), \end{array}$$

where all *A, B and C* ∈ Ω, *A* ≠ ϕ, *m*_1_ ⊕ *m*_2_(ϕ) = 0. Here, κ is the degree of conflict, called the conflict coefficient, between *m*_1_ and *m*_2_, and it is calculated by the sum of the products *m*_1_(*B*)*m*_2_(*C*) for all focal elements *B* in *m*_1_ and *C* in *m*_2_ with *B* ∩ *C* = ϕ. The larger the value of κ is, the more the two sources conflict. When κ = 1, it implies that these two pieces of evidence are in complete logical contradiction. Here κ also functions as a normalization constant to let the joint BPA observe the property ∑_*A*∈Ω_ *m*(*A*) = 1 in D–S theory. Dempster's rule of combination is associative and commutative; hence, the overall BPA resulting from the combination of all decision agents/classifiers is defined as

(14) $$\begin{array}{r} m_{total}=m_{1}⊕m_{2}⊕\ldots ⊕m_{j}, \end{array}$$

where *j* is the total number of classifiers and *m*_*total*_ aggregates information from all the individual sources and represents the aggregated mass function after the D–S fusion process.

After combining all the BPAs, the overall BPA is transformed into pignistic probability that focuses on singletons to make a decision.

(15) $$\begin{array}{r} p_{pig}\left(\left\{a\right\}\right)=\sum_{a\in B}\frac{m\left(B\right)}{\left|B\right|}\;\forall a\in \Omega \end{array}$$

where |*B*| is the number of singleton elements in set *B*. The hypothesis (the class) with the maximum pignistic probability is chosen as the predicted class of the sample in the test data.

The proposed method quantifies evidence from each classifier and assigns basic probabilities to both single and compound hypotheses. Our approach uses the ROU to define a compound hypothesis, which helps to avoid overestimation of uncertainty compared to the complementary approach. In addition, to exploit the characteristics associated with different sources, we employ a weighted regulatory architecture to assign mass values to single and compound hypotheses. We use a training mechanism to find the appropriate weights (i.e., α and β) for different types of evidences. To further strengthen the FDMF, we can use different classification approaches to determine BPAs. However, the appropriate selection of classification approaches is not the main focus of this study.

## Experiment

Myriad data can be acquired from many different sensors, and each sensor has its own limitations and associated uncertainty. Therefore, fused information from such sources/classifiers can be expected to yield better decisions by reducing the overall uncertainty. This study conducted an RSVP experiment to demonstrate how the HMA system integrates an RSVP BCI with computer vision in an image-labeling domain and to examine how the FDMF can benefit joint human-autonomy performance. Because different levels of uncertainty are embedded within disparate human and machine knowledge, we expect that the FDMF would be able to exploit these uncertainties to obtain superior decisions.

### Rapid serial visual presentation paradigm

**Figure 4:**
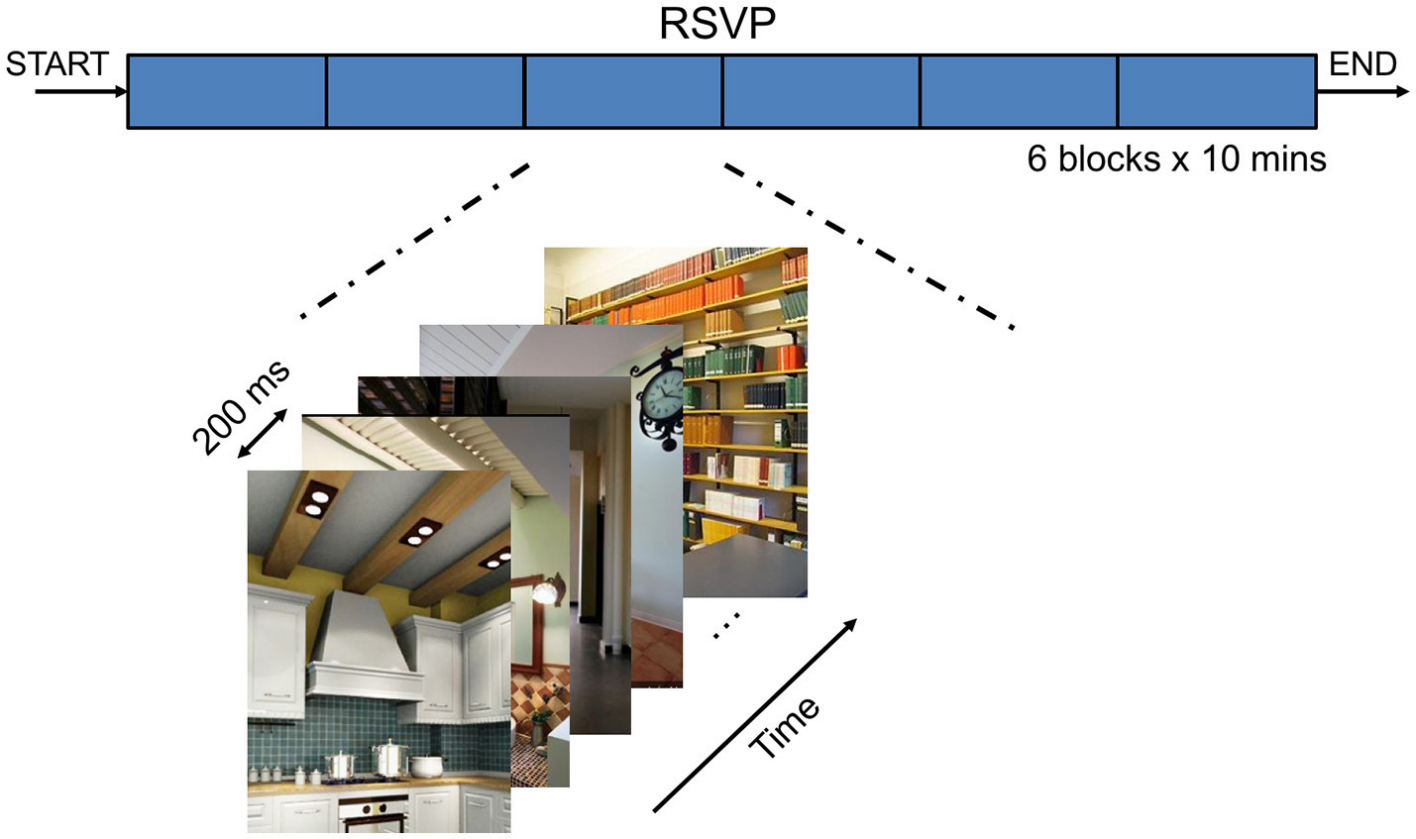
The Rapid Serial Visual Presentation (RSVP) task.

During the RSVP triage task, participants were presented with a continuous sequence of natural scenes. The RSVP paradigm is shown in Figure 4. The RSVP task consisted of the serial presentation, on a computer monitor, of 512 × 662-pixel color photographs of indoor and outdoor scenes at a rate of 5 Hz. Therefore, the time interval between two consecutive photographs is 200 ms. For each single photograph, the EEG epoch was segmented lasting from 500 to 1,000 ms to extract characteristics of the related brain activities. The entire sequence of images was presented in six blocks of 3,000 images each; each presentation block lasted 10 min. The scenes contained only inanimate objects, and the images were manually cropped. Five classes of objects could be designated as targets: stairs, containers, posters, chairs, and doors. Some images contained designated target objects; others did not. The order of images containing target classes was randomly selected for each participant during blocks 1–5. However, block 6 always used the same target object as block 1. Moreover, because some images contained multiple targets (e.g., chair and container) they were repeatedly presented in subsequent blocks; however, these were considered as different stimuli depending on the target context of the current block. Because no blocks have multiple target classes, duplicate target images were never shown together in the same block. Prior to the presentation of each block of images, participants were informed about objects class that comprised the targets. Participants were required to press a button when they saw a target object in the RSVP stream.

### Participants

Seventeen healthy adults participated in the RSVP task, in which they attempted to identify sparse target images within a continuous stream of images presented at 5 Hz. All the participants were recruited through an online advertisement. According to self-reports, no subject had a history of neurological, psychiatric or addictive disorders, and no subject had taken anti-psychotic or other relevant psychoactive drugs prior to participation. All the participants were required to read and sign an informed consent form prior to participation, and all experimental procedures were in compliance with federal and Army regulations (U.S. Department of the Army, 1990; U.S. Department of Defense Office of the Secretary of Defense, 1999). The Army Research Laboratory Institutional Review Board, Maryland, USA, approved the study. The investigators adhered to Army policies for the protection of human subjects (U.S. Department of the Army, 1990). A more detailed description of the experimental protocols and the results are available in Touryan et al. (2013, 2014). To ensure proper evaluations of their performances, the participants attended a pre-test session to verify that none were afflicted with any sickness caused by the RSVP experiment.

### Cross-validation and assessment

The empirical gains for this FDMF were established and cross-validated using standard machine learning approaches described below. The human responses included the RT of the button press and the output of the three EEG classifiers. The CV responses for each target were generated by the CV trained on that target. For the simulations, all the classifiers were trained and tested on independent data using a six-fold cross-validation scheme. The data were separated into six equally sized blocks (corresponding to the six blocks of RSVP presented to the participant), and these blocks were used in six-fold cross-validation (i.e., five blocks of the data are selected as training dataset, while the remaining block serves as test data). This process was repeated six times, and the average of the six runs was then used for comparisons. Note that, although these particular simulations involved *post-hoc* human-autonomy decision integration, all the methods were amenable to real-time implementation.

Performance was quantified using the area (Az) under the ROC curve, which characterizes the performance of a binary classifier as a function of the ratio of the true positive rate to the false positive rate. A one-way ANOVA showed a significant difference in the performance among different fusion models. Subsequent paired *t*-tests, corrected for multiple comparisons using the false discovery rate (FDR), indicated a significantly improved performance of FDMF compared with the existing fusion models that we have considered.

## Results

In this study, the efficacy of the proposed FDMF in human-machine information fusion is demonstrated in an RSVP paradigm, in which targets are recognized from a continuous sequence of natural scenes. To establish multi-view estimation, the simultaneously recorded EEG and behavioral signals are used to build an ensemble of three neural classification algorithms (i.e., HDCA, XD+BLDA, and CSP+BLDA) and a behavioral classifier (i.e., BTN). Furthermore, the video streams are also simultaneously analyzed using the CV technology (i.e., TAG) for target detection. As shown in Figure 2, the proposed HMA system in this study exploits a two-stage hierarchical mechanism for information integration, including classifier fusion and multi-modality (human and machine) knowledge fusion. For each modality collected in this study, different classifiers are designed, and then, FDMF is used to aggregate the evidence from these classifiers. The FDMF aims to leverage recent advances in human sensing to dynamically integrate human and autonomous agents based on their individual characteristics.

Table 1 depicts the classification results of different comparative models obtained by six-fold cross-validation across 17 subjects; the best performance is shown in bold face. The average Azs of the three neural classifiers (HDCA, XD+BLDA, and CSP+BLDA) using EEG signals alone are 0.645 ± 0.050, 0.711 ± 0.045, and 0.616 ± 0.049, respectively, and the average Az of the behavioral classifier (BTN) is 0.640 ± 0.066. In contrast, the classification performance of the TAG classifier is 0.828 ± 0.020. Further, the results of different fusion strategies using the FDMF are shown in Table 2. The average performance from integrating all the neural classifiers is 0.729, and the average performance of human decision based on the neural and behavioral classifiers is 0.769. However, the overall performance of the HMA system based on the human and machine knowledge achieves 0.8872. This result demonstrates that the HMA system achieves the highest Az compared with the other single classifiers before the fusion stage (Table 2). The best result achieved is an Az of 0.896 while using the combination of the best neural classifier (XD+BLDA), behavioral classifier (BTN), and CV algorithm (TAG) - this combination can improve the performance of the HMA system. These results suggest that the use of the proposed FDMF for integration of human and machine knowledge can effectively enhance the performance of HMA systems during RSVP tasks.

**Table 1:** Area (Az) under the receiver operating characteristic (ROC) curve of comparative models.

	**Human**				**Machine**	**Fusion method**			
	**HDCA**	**XD+BLDA**	**CSP+BLDA**	**BTN**	**TAG**	**LR**	**FI**	**WAS**	**FDMF**
Mean	0.645	0.711	0.616	0.640	0.828	0.844	0.855	0.860	**0.896**
Std	0.050	0.045	0.049	0.066	0.020	0.020	0.015	0.020	**0.024**
StatisticalTest	+	+	+	+	+	+	+	+	N/A

*H, HDCA; X, XD+BLDA; C, CSP+BLDA*.

*The symbol + refers to a situation in which the average accuracy of the proposed FDMF is significantly different compared to a comparative model suggested by post-hoc comparisons (Paired t-test, p < 0.05). Bold value indicates the best performance*.

**Table 2:** Different fusion strategies using the fuzzy decision-making fuser (FDMF).

	**Combination**			**Az**
Type 1	Human (H+X+C)			0.729
Type 2	Human (H+X+C)	Behavior (BTN)		0.769
Type 3	Human (H+X+C)		Machine (TAG)	0.859
Type 4		Behavior (BTN)	Machine (TAG)	0.890
Type 5	Human (H+X+C)	Behavior (BTN)	Machine (TAG)	0.887
**Type 6**	**1st Human (X)**	**Behavior (BTN)**	**Machine (TAG)**	**0.896**
Type 7	2nd Human (H)	Behavior (BTN)	Machine (TAG)	0.889
Type 8	3rd Human (C)	Behavior (BTN)	Machine (TAG)	0.889

*In column 2, H = HDCA, X = XD+BLDA, and C = CSP+BLDA. Bold value indicates the best performance*.

This study also compared the FDMF with existing multi-modal information fusion approaches: linear regression (LR), fuzzy integral (FI), and weighted averages (WAS). As shown in Table 1, the FDMF outperforms these other methods. To determine whether the improvement resulting from employing FDMF was statistically significant, the experimental results were subjected to analysis of variance (paired *t*-tests and FDR-adjusted *p* < 0.05) for each comparative model. The results of the *post-hoc* paired *t*-tests are shown in Table 1. The models marked by a plus (“+”) sign showed significant differences from the performance achieved by the FDMF. These results show that all the compared models are significantly different (all *p* < 0.05), indicating that FDMF is the best-performing fusion approach in this study.

## Conclusion

Developing novel techniques to combine multi-modal information to produce highly accurate classification is vital. This study proposes an HMA system that can simultaneously aggregate human and machine knowledge to identify target objects in an RSVP task. To better integrate the human and machine decisions and to deal with the uncertainty associated with decisions by each agent, this study has used the FDMF, which is based on D–S theory, to provide a natural adaptive framework for evidence-based inference. The FDMF exploits a novel compound model to determine BPA that combines both generative-type and discriminative-type approaches. Unlike most other methods, the proposed method applies a plausible mathematical structure to determine evidence weights. In addition, a novel belief assignment that can directly represent uncertainties and imprecision during the classification process is employed to capture uncertainties between classes. The results of these experiments show that the proposed fusion model produces reliable and robust decisions compared to those produced by single agents, i.e., human or machine agents, before the fusion stage. The major benefit gained from the FDMF is that the system can furnish information of high quality concerning, possibly, certain aspects of the environment that cannot be sensed directly by any individual sensor operating independently. The performance of the proposed HMA system indicates that the FDMF model for integrating human and machine knowledge is both reasonable and effective.

The main limitation of our system is that it is computationally a bit more expensive than use of simple aggregation techniques like voting or individual classifier. Moreover, in general, BPA can (and sometimes need to) be assigned to focal elements with cardinality more than two. But this will increase the computational complexity significantly. Another weakness of our study is that here we did not tune the parameters (mean and spread) of the membership functions. The system performance can further be improved if we tune them, although the associated optimization model would not be simple. We keep this for our future investigation.

## Author contributions

YL mainly worked on developing the structure of the human-machine system and the fuzzy decision-making fuser algorithm. NP mainly worked on designing the training algorithm of the fusion system. AM majorly worked on data collection and decision-making fuser algorithm. CL and YW mainly worked on developing the brain-computer interface used in the human-machine system.

### Conflict of interest statement

The authors declare that the research was conducted in the absence of any commercial or financial relationships that could be construed as a potential conflict of interest.
